# Between-rater reliability for using radar technology to quantify maximal horizontal deceleration performance in NCAA division 1 American football and female lacrosse athletes

**DOI:** 10.3389/fspor.2024.1384476

**Published:** 2024-07-01

**Authors:** Nicolas M. Philipp, Ben McKay, Ethan Martin, Dimitrije Cabarkapa, Andrew C. Fry, Jordan Troester

**Affiliations:** ^1^ Jayhawk Athletic Performance Laboratory—Wu Tsai Human Performance Alliance, University of Kansas, Lawrence, KS, United States; ^2^ Centre for Medical and Exercise Physiology, School of Medicine, University of Wollongong, Wollongong, NSW, Australia; ^3^ Athletic Department, University of Oregon, Eugene, OR, United States

**Keywords:** deceleration, reliability, sprint, American football, lacrosse

## Abstract

**Introduction:**

With recent increases in the popularity of studying the physical construct of horizontal deceleration performance in team-sport athletes, the aim of the present study was to assess the inter-rater and intra-rater reliability of processing and quantifying horizontal deceleration ability using radar technology.

**Methods:**

Data from 92 NCAA Division 1 athletes from two different athletic teams (American football and Lacrosse) were used for the present investigation. All athletes performed two trials of the modified acceleration to deceleration assessment (ADA), which consisted of a maximal 10 m sprint acceleration, followed by a rapid deceleration. Four individual raters manually processed raw, radar-derived instantaneous velocity data for the ADA, and an automated script was used to calculate metrics of interest.

**Results:**

Primary study findings suggest moderate to excellent levels of agreement (ICC = 0.56–0.91) for maximal horizontal deceleration metrics between the four individual raters. The intra-rater analyses revealed poor to excellent consistency (ICC = 0.31–0.94) between ADA trials, with CV%'s ranging from 3.1% to 13.2%, depending on the respective metric and rater.

**Discussion:**

Our data suggests that if a foundational understanding and agreement of manual data processing procedures for radar-derived data is given between raters, metrics may be interpreted with moderate to excellent levels of confidence. However, when possible, and when using the Stalker ATS radar technology, authors recommend that practitioners use one trained individual to manually process raw data. Ideally, this process should become fully automated, based on selected filters or algorithms, rather than the subjectivity of the rater.

## 1 Introduction

Sport Science is a rapidly growing field that applies scientific methods to sport settings to improve performance and athlete wellbeing. At the center of this profession is the sport scientist, who is tasked with duties related to performance enhancement programming, testing and profiling, and the monitoring of training load and injury trends (1). With the rapid expansion of technology is sports (2) it is now more important than ever for sport scientists to take ownership of their data by ensuring that the technologies and collection/analysis methods have been critically evaluated (1). Currell and Jeukendrup proposed three factors that contribute to a good performance test: (i) validity; (ii) reliability; and (iii) sensitivity (3). Reliability refers to the reproducibility of the values of a test (4) and can be influenced by variation in performance by the test subject, variation in the test methods, and variation in measurement of testing equipment (5). In a sports scientist's desire to adopt new technologies and assessment methods, the first step of this process should be an evaluation of reliability.

While sports scientists have been quantifying athletes speed (horizontal acceleration and maximal velocity) for quite some time, assessment of horizontal deceleration performance has only recently become more prevalent (6–12). Given the importance of deceleration to the demands of numerous sports, quantifying athletes' ability to reduce velocity and whole-body momentum may have significant implications for health and performance (10). Research has shown that in some field-based sports such as soccer and rugby athletes perform more high-intensity decelerations compared to accelerations, and that these high-intensity decelerative actions may have a profound impact on competition-related muscle damage, fatigue and recovery (7, 13–15). Both American football and lacrosse are multi-directional sports in which athletes are frequently exposed to high intensity deceleration demands in order to generate separation from defenders, perform cutting motions or complete rapid changes in direction (16–20). During maximal horizontal decelerations, the braking steps exhibit a distinct ground reaction force profile characterized by high-impact peak forces and loading rates, in some cases up to six times body mass, which is nearly three times higher than maximal horizontal acceleration (6). These forces and loading rates must be met with appropriate neuromuscular (e.g., braking force attenuation), coordinative, and skill-related qualities to efficiently reduce whole body momentum (6). With potential implications for performance (e.g., generation of space between players, change of direction ability), and health (e.g., injury risk reduction) (10), it is important for sport science practitioners to possess the means to quantify deceleration ability in a reliable and valid fashion.

According to Harper et al., prior to 2020 only a small number of studies have tried to quantify and highlight the importance of horizontal deceleration performance (9, 21–24), likely due to a lack of methods, tests, and technologies to effectively quantify horizontal deceleration. Radar and laser devices have historically been used to assess athlete's horizontal sprint acceleration abilities (25), and more recent research has used this same technology to measure horizontal deceleration. Harper et al. proposed the use of radar-derived instantaneous velocity to calculate different metrics of interest during a novel acceleration to deceleration task, termed the acceleration to deceleration ability assessment (ADA) (8). This test requires athletes to maximally accelerate over 20 m, followed by a rapid and maximal deceleration, and backpedal to the 20-m marker (8). Based on the dimensions of the playing field, recent research has modified this protocol to include a 10-m acceleration instead of a 20-m acceleration (11, 12). While different studies have reported reliability and variability scores in their results, to the authors knowledge, only one study has investigated the inter-, and intra-day reliability of several kinetic and kinematic deceleration metrics derived from a radar device (8). In this study the majority of ADA-derived metrics showed good intra-day reliability and were sufficiently sensitive to detect small-to-moderate worthwhile changes in deceleration performance. Further, only kinetic variables had good inter-day reliability, and were adequately able to detect moderate worthwhile changes in deceleration performance after a single familiarization session. The proposed ADA methods contain two steps that require manual processing of raw data; (i) manually deleting all data recorded before the start of the sprint and following the end of the deceleration phase, and (ii) manually removing unexpected high and low data points on the velocity-time curve that were likely caused by segmental movements (e.g., arms) of the participants while running (8). These steps which contain no clear thresholds for eliminating data points may leave room for methodological variability. Previous studies reporting the reliability of ADA assessments used a single rater to manually process raw data (26), and the introduction of multiple individuals to process the raw data may add methodological variability making it difficult to compare results between studies, different populations or athlete groups, as well as comparing results over time within the same athletes.

Therefore, the primary aim of the study was to investigate the between-rater agreement of four raters for processing and quantifying ADA-derived acceleration and deceleration metrics using radar technology. The secondary aim was to investigate the intra-rater reliability and variability (i.e., within-session) between the two trials each athlete performed for all metrics of interest, and across all raters. Authors speculated possible disagreement between raters based on the subjective nature of the initial data treatment process, which could negatively impact the usage and interpretation of results.

## 2 Materials and methods

### 2.1 Subjects

Data from a total of 72 male, NCAA Division 1, collegiate American football players (height = 184 ± 7 cm, weight = 91.8 ± 13.3 kg), and 20 female, NCAA Division 1, collegiate lacrosse players (height = 166 ± 7 cm, weight = 63.2 ± 7.0 kg) were used for the present study. For their data to be included in this study, subjects had to be an active member of either the American football or lacrosse team at the University and had to be medically cleared by the respective sports medicine staff for full sport participation. All athletes provided written consent for their deidentified data to be used for research purposes, as approved by the University's institutional review board.

### 2.2 Experimental design

This study aimed to investigate the between-rater agreement for measuring acceleration and deceleration performance quantified through the ADA test using a single-session design. Additionally, within-session (i.e., test-retest) reliability and variability statistics were calculated. A sample of 92 NCAA Division 1 athletes from two athletic teams (Men's American football & Women's Lacrosse) performed two trials of the ADA. Data were collected by the same individual, manually processed by four different individuals, and metrics of interest were calculated by a single individual. All raters presented with similar levels of familiarity with the radar technology, and raters had a formal meeting prior to the start of the study to review raw data processing guidelines for assessing acceleration and deceleration data using radar technology (8, 26). Further, all athletes were familiar with the respective procedures through exposure as part of their strength and conditioning program.

### 2.3 Acceleration deceleration assessment

Methods for collection of acceleration and deceleration data using the ADA test were adapted from previous research (8, 11, 12). Instead of a 20-m sprint acceleration as first proposed by Harper et al., subjects in this study maximally accelerated over 10-yd (9.14 m), followed by a rapid deceleration coming to a stop. Athletes started in a two-point, staggered stance prior to initiating the sprint, and were instructed to maximally accelerate over 9.14-m, with the 9.14-m mark being identified with cones. Immediately after crossing the 9.14-m mark, athletes were instructed to rapidly decelerate, and come to a stop as fast as possible. Following the deceleration phase, athletes backpedaled back to the 9.14-m mark to create a clear change in velocity on the velocity-time graph, to aid in later treatment of raw data. Athletes performed a total of two trials, with 3 min of passive rest between each trial. If athletes were visually observed to slow down prior to the 9.14-m mark, or significantly after it, the trial was repeated following 3 min of passive rest.

In line with previous research, instantaneous velocity was measured during the entire ADA test, using a tripod mounted radar device (Stalker ATS II, Applied Concepts, Inc., Dallas, TX, USA) that was placed approximately 5 m behind the start line, with a height that was in line with the athletes' center of mass. The radar device sampled at a frequency of 47 Hertz. To allow for the radar to capture instantaneous velocity while the athlete is moving away from and towards the radar, the target direction on the radar was set to “both”. Following suggestions by Harper et al., when the athlete was in the stationary two-point stance, data recording was started using the “any key” feature within the Stalker ATS software, and a verbal instruction of “whenever you are ready” was given to the athlete (8). Data collection was terminated in a similar fashion after the athlete had backpedaled back to the 9.14-m marker.

### 2.4 Radar data analysis

**Figure 1:**
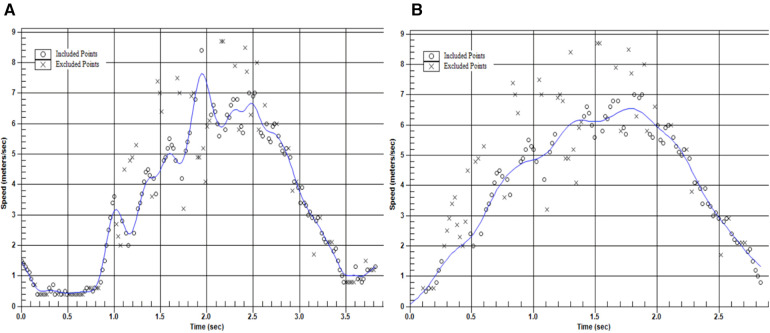
Example unprocessed (panel **A**) and processed (panel **B**) velocity-time trace for the ADA test used in our study.

As suggested in previous research, all data was manually processed in the graph mode editor within the Stalker ATS software (8, 26). These procedures were adapted from Simperingham et al. and as mentioned in the introduction involved (i) deleting all data recorded before the start of the sprint and following the termination of the deceleration phase, (ii) nominating all trials to be “acceleration runs” thereby forcing the start of the velocity-time curve through the zero point, (iii) applying a digital fourth order, zero lag Butterworth filter, and (iv) manually removing unexpected high and low data points on the velocity-time curve that were likely caused by segmental movements of the athletes while sprinting (26). Figure 1 presents a visualization of an example unprocessed and a processed velocity-time curve for the ADA test. This figure only presents instantaneous velocity-time data for the acceleration and deceleration phase of the ADA, not the backpedal following the maximal deceleration. Four raters separately completed these manual data processing procedures. Following manual processing, data were exported into the RStudio software (Version 1.4.1106) for further analysis. In line with previous work, the deceleration phase was defined as starting immediately following the athletes reaching peak velocity (Max Velo) and terminated at the point of lowest velocity following peak velocity (Min Velo) (8). Furthermore, in line with previous work, the deceleration phase was divided into an early and a late deceleration phase, using 50% of Max Velo during deceleration as the respective cut-off point (8). All metric calculations were derived from instantaneous velocity and time, with instantaneous horizontal acceleration and deceleration being calculated between each data point captured across the entire acceleration and deceleration phase respectively. Metrics of interest and respective metric calculations were adapted from previous research (8) and may be found in Table 1. The manual processing procedures were carried out by each rater separately, while metric calculations in RStudio were performed by the same researcher via an automated script. For the between-rater analysis, the athlete's mean of their two trials were used, while the within-session (i.e., test-retest) reliability and variability statistics were calculated between the two trials performed. Previous research has suggested sufficient within-, and between-day reliability for metrics derived from the ADA test (8), however, between-rater reliability for the manual data processing procedures described in this paragraph have yet to be explored.

**Table 1:** ADA metrics and respective definitions.

Metric (unit)	Definition|calculation
Average acceleration (m/s^2^)	Average of all instantaneous data points captured across the acceleration phase
Maximal acceleration (m/s^2^)	Maximum of all instantaneous data points captured across the acceleration phase
Maximal approach velocity (m/s)	Maximal velocity attained immediately prior to the deceleration phase
Average deceleration (m/s^2^)	Average of all instantaneous data points captured across the deceleration phase
Maximal deceleration (m/s^2^)	Maximum of all instantaneous data points captured across the deceleration phase
Time to stop (s)	Time from the start of the deceleration phase to the end of the deceleration phase
Deceleration distance (m)	Distance from the start of the deceleration phase to the end of the deceleration phase
Average early deceleration (m/s^2^)	Average of all instantaneous data points captured across the early deceleration phase
Average late deceleration (m/s^2^)	Average of all instantaneous data points captured across the late deceleration phase

### 2.5 Statistical analysis

Descriptive information for this study is presented as means and standard deviations. All data were explored for normality using a Shapiro-Wilk test. To investigate the between-rater agreement, a two-way mixed effects model intraclass correlation coefficient analysis ICC_2_, _k_(agreement) was conducted using the “irr” package in RStudio (Version 1.4.1106), and interpreted where <0.50 was deemed poor reliability, 0.50–0.74 was deemed moderate reliability, 0.75–0.90 was deemed good reliability, and >0.90 was deemed excellent reliability (27). Further, between-rater coefficient of variation percentages (CV%) were calculated by dividing the standard deviation of the ADA trials between raters, by the mean of the ADA trials between raters and multiplying it by 100 to generate a percentage. CV%'s were interpreted as “excellent” if less than 10% (CV < 10%) as recommended in previous research (28, 29). Similarly, to investigate within-test consistency (i.e., intra-rater reliability) for each rater between the two ADA trials performed, ICC_2_, _k_(consistency) and coefficient of variation percentages (CV%) were calculated. Within-test consistency was analyzed and reported for each rater respectively. Lastly, for both inter-rater, and intra-rater analyses, standard errors of measurement (SEM) were calculated following previously established guidelines (30). The SEM provides an absolute index of reliability and has the same units as the metric of interest, while the ICC is unitless (30). All data were analyzed in the RStudio software (Version 1.4.1106), and statistical inferences were made at the *p* ≤ 0.05 significance level.

## 3 Results

**Figure 2:**
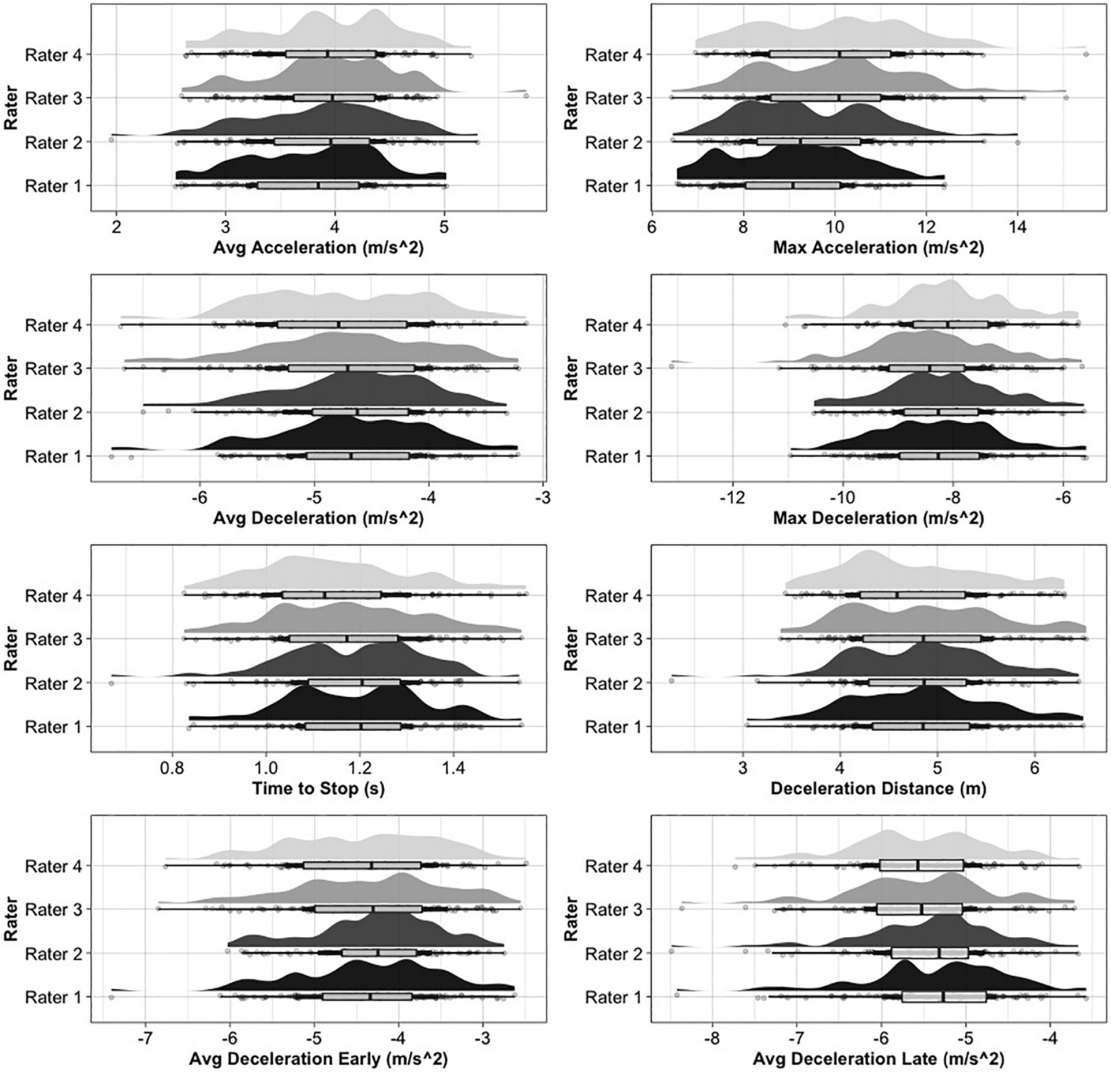
Raincloud plots showing boxplots, half-violin plots, and data jitter to visualize agreement between raters for selected deceleration metrics.

Sport-specific descriptive information presented as means and standard deviations across all raters may be found in Table 2. Inter-rater reliability statistics may be found in Table 3. The inter-rater analysis revealed ICCs ranging from moderate to excellent agreement across all ADA-derived metrics, with CV%'s ranging from 3.5 to 7.8. F-values ranged from 6.07 to 7.8 In line with the secondary aim of the study, Table 4 presents intra-rater (i.e., within-session) reliability and variability statistics across all four raters. Intra-rater ICCs ranged from poor to excellent consistency, while CV%'s ranged from 3.1 to 13.2. F-values ranged from 1.87 to 22.0. SEM values displaying inter-, and intra-rater variability in the same units as the variable of interest may be found in Tables 3, 4, respectively. Figure 1 displays raincloud plots showing boxplots, half-violin plots, and data jitter to visualize agreement between raters for selected deceleration metrics, while Figure 2 visualizes the inter-rater ICCs plus respective confidence intervals.

**Table 2:** Sport-, and position-specific descriptive statistics for ADA-derived metrics of interest (x¯ ± SD across all raters).

American football (*n* = 72)									
	Avg ACC (m/s^2)^	Max ACC (m/s^2^)	Max Velo (m/s)	Avg DEC (m/s^2^)	Max DEC (m/s^2^)	TTS (s)	DEC Dist (m)	Avg DEC_E_ (m/s^2^)	Avg DEC_L_ (m/s^2^)
CB (*n* = 8)	4.29 ± 0.44	10.0 ± 1.46	7.02 ± 0.26	−5.06 ± 0.61	−8.97 ± 0.99	1.14 ± 0.15	5.10 ± 0.86	−4.74 ± 0.85	−6.02 ± 1.09
ILB (*n* = 9)	4.44 ± 0.45	10.7 ± 1.84	6.66 ± 0.28	−4.62 ± 0.45	−8.75 ± 0.51	1.26 ± 0.11	5.24 ± 0.51	−4.24 ± 0.56	−5.28 ± 0.83
OLB (*n* = 8)	3.93 ± 0.35	10.6 ± 0.97	6.57 ± 0.17	−4.76 ± 0.55	−9.04 ± 1.08	1.20 ± 0.14	4.81 ± 0.80	−4.51 ± 0.73	−5.29 ± 0.61
QB (*n* = 5)	3.73 ± 0.20	8.57 ± 1.37	6.26 ± 0.27	−4.52 ± 0.77	−8.31 ± 0.36	1.13 ± 0.08	4.60 ± 0.58	−4.25 ± 0.92	−5.20 ± 0.55
RB (*n* = 9)	4.24 ± 0.61	10.3 ± 1.39	6.56 ± 0.38	−4.72 ± 0.54	−8.46 ± 0.79	1.13 ± 0.16	4.86 ± 0.87	−4.38 ± 0.70	−5.55 ± 0.60
SAF (*n* = 9)	4.07 ± 0.38	9.65 ± 1.43	6.73 ± 0.32	−5.18 ± 0.74	−8.85 ± 0.76	1.11 ± 0.15	4.71 ± 0.82	−4.83 ± 0.92	−5.87 ± 0.75
SP (*n* = 24)	3.81 ± 0.40	9.63 ± 1.03	6.35 ± 0.25	−4.30 ± 0.59	−7.66 ± 1.09	1.27 ± 0.18	5.03 ± 0.91	−3.96 ± 0.66	−4.98 ± 0.93
TE (*n* = 5)	3.93 ± 0.50	9.18 ± 1.21	6.31 ± 0.22	−4.58 ± 0.65	−8.27 ± 0.97	1.17 ± 0.16	4.63 ± 0.70	−4.17 ± 0.65	−5.34 ± 0.93
WR (*n* = 11)	4.24 ± 0.32	10.4 ± 1.74	6.90 ± 0.17	−5.25 ± 0.64	−8.57 ± 0.85	1.08 ± 0.13	4.58 ± 0.55	−5.20 ± 0.70	−5.43 ± 0.86
Women's lacrosse (*n* = 20)									
	Avg ACC (m/s^2)^	Max ACC (m/s^2^)	Max Velo (m/s)	Avg DEC (m/s^2^)	Max DEC (m/s^2^)	TTS (s)	DEC Dist (m)	Avg DEC_E_ (m/s^2^)	Avg DEC_L_ (m/s^2^)
ATT (*n* = 5)	2.94 ± 0.31	8.32 ± 1.67	5.93 ± 0.20	−4.08 ± 0.43	−6.89 ± 1.64	1.24 ± 0.17	4.84 ± 0.58	−3.54 ± 0.37	−5.31 ± 0.81
DEF (*n* = 5)	3.10 ± 0.16	8.16 ± 1.03	5.89 ± 0.12	−4.03 ± 0.49	−7.29 ± 0.55	1.27 ± 0.13	4.92 ± 0.63	−3.45 ± 0.49	−5.30 ± 0.67
GK (*n* = 1)	2.94 ± 0.03	9.77 ± 0.29	5.97 ± 0.03	−3.97 ± 0.08	−6.59 ± 0.01	1.32 ± 0.02	4.66 ± 0.11	−3.73 ± 0.14	−4.32 ± 0.03
MF (*n* = 9)	3.14 ± 0.37	8.40 ± 0.76	6.03 ± 0.26	−4.46 ± 0.55	−7.43 ± 0.63	1.16 ± 0.13	4.61 ± 0.66	−3.88 ± 0.63	−5.73 ± 0.64

CB, corner back; ILB, inside linebacker; OLB, outside linebacker; QB, quarterback; RB, running back; SAF, safety, SP, special teams player; TE, tight end; WR, wide receiver; ATT, attacker; DEF, defender; GK, goalkeeper; MF, midfielder.

**Table 3:** Inter-rater reliability statistics (between-rater ICC).

Metric	Agreement_ICC_	95% CI	F-value	SEM	Classification	CV%
Avg ACC (m/s^2^)	0.85	0.80–0.89	26.2	0.22	Good	4.6
Max ACC (m/s^2^)	0.75	0.62–0.84	17.5	0.69	Good	6.9
Max Velo (m/s)	0.91	0.88–0.94	45.7	0.12	Excellent	1.2
Avg DEC (m/s^2^)	0.79	0.72–0.84	16.0	0.32	Good	5.3
Max DEC (m/s^2^)	0.83	0.78–0.88	22.9	0.43	Good	3.5
TTS (s)	0.67	0.59–0.75	9.61	0.09	Moderate	6.0
DEC Dist (m)	0.56	0.46–0.66	6.07	0.49	Moderate	7.5
Avg DEC_E_ (m/s^2^)	0.73	0.65–0.80	11.7	0.45	Moderate	7.8
Avg DEC_L_ (m/s^2^)	0.86	0.81–0.90	27.1	0.30	Good	4.1

Avg, average; Max, maximal; velo, velocity; ACC, acceleration; DEC, deceleration; TTS, time to stop; Dist, distance; SEM, standard error of measurement.

**Table 4:** Raters 1 to 4 intra-rater reliability and variability statistics (i.e., test-retest ICC, SEM and CV%) .

Metric	Consistency_ICC_	95% CI	F-value	SEM	Classification	CV%
Rater 1						
Avg ACC (m/s^2^)	0.94	0.86–0.94	20.6	0.17	Excellent	4.3
Max ACC (m/s^2^)	0.50	0.31–0.64	2.97	1.10	Moderate	9.5
Max Velo (m/s)	0.67	0.53–0.77	5.02	0.26	Moderate	3.4
Avg DEC (m/s^2^)	0.53	0.36–0.67	3.29	0.53	Moderate	8.9
Max DEC (m/s^2^)	0.52	0.34–0.66	3.12	0.88	Moderate	7.9
TTS (s)	0.31	0.10–0.50	1.91	0.14	Poor	11.3
DEC Dist (m)	0.35	0.15–0.53	2.08	0.73	Poor	13.2
Avg DEC_E_ (m/s^2^)	0.63	0.48–0.75	4.4	0.60	Moderate	12.4
Avg DEC_L_ (m/s^2^)	0.33	0.13–0.51	2.0	0.88	Poor	11.9
Rater 2						
Avg ACC (m/s^2^)	0.90	0.85–0.93	19.0	0.20	Excellent	4.1
Max ACC (m/s^2^)	0.46	0.27–0.62	2.72	1.41	Poor	10.6
Max Velo (m/s)	0.67	0.54–0.78	5.14	0.26	Moderate	3.1
Avg DEC (m/s^2^)	0.64	0.49–0.75	4.59	0.50	Moderate	8.7
Max DEC (m/s^2^)	0.38	0.18–0.55	2.22	1.19	Poor	8.0
TTS (s)	0.47	0.28–0.62	2.77	0.14	Poor	10.1
DEC Dist (m)	0.44	0.25–0.60	2.58	0.70	Poor	11.9
Avg DEC_E_ (m/s^2^)	0.70	0.57–0.79	5.6	0.56	Moderate	11.5
Avg DEC_L_ (m/s^2^)	0.30	0.09–0.49	1.87	0.88	Poor	12.1
Rater 3						
Avg ACC (m/s^2^)	0.90	0.85–0.93	18.9	0.20	Excellent	4.2
Max ACC (m/s^2^)	0.72	0.59–0.81	6.01	0.96	Moderate	7.5
Max Velo (m/s)	0.60	0.43–0.72	3.93	0.30	Moderate	3.4
Avg DEC (m/s^2^)	0.73	0.60–0.82	6.30	0.41	Moderate	6.9
Max DEC (m/s^2^)	0.56	0.39–0.69	3.54	0.84	Moderate	7.9
TTS (s)	0.44	0.24–0.60	2.58	0.14	Poor	10.9
DEC Dist (m)	0.47	0.28–0.62	2.74	0.66	Poor	12.1
Avg DEC_E_ (m/s^2^)	0.75	0.64–0.83	7.10	0.49	Good	9.3
Avg DEC_L_ (m/s^2^)	0.32	0.11–0.50	1.95	0.82	Poor	11.3
Rater 4						
Avg ACC (m/s^2^)	0.91	0.87–0.94	22.0	0.20	Excellent	4.3
Max ACC (m/s^2^)	0.57	0.40–0.70	3.64	1.05	Moderate	8.6
Max Velo (m/s)	0.53	0.35–0.67	3.23	0.36	Moderate	3.6
Avg DEC (m/s^2^)	0.59	0.43–0.72	3.86	0.45	Moderate	7.7
Max DEC (m/s^2^)	0.53	0.36–0.67	3.28	0.83	Moderate	7.7
TTS (s)	0.36	0.16–0.54	2.14	0.14	Poor	11.1
DEC Dist (m)	0.41	0.22–0.58	2.41	0.67	Poor	13.1
Avg DEC_E_ (m/s^2^)	0.61	0.43–0.73	4.06	0.52	Moderate	10.3
Avg DEC_L_ (m/s^2^)	0.34	0.13–0.52	2.02	0.84	Poor	12.2

Avg, average; Max, maximal; Velo, velocity; ACC, acceleration; DEC, deceleration; TTS, time to stop; Dist, distance; SEM, standard error of measurement.

## 4 Discussion

The primary aim of this study was to investigate the between-rater reliability/agreement for quantifying maximal horizontal deceleration performance in collegiate NCAA Division 1 athletes using radar technology. Four individual raters manually processed raw velocity-time data from an assessment quantifying maximal acceleration and deceleration using suggestions from previous research reports (8). An automated script was used to calculate metrics of interest, which were based on the manually processed velocity-time data. A secondary study aim was to determine the intra-rater (i.e., within-session) reliability and variability of all ADA-derived metrics.

The inter-rater reliability analyses reported ICC's ranging from moderate to excellent agreement, with TTS, DEC Dist, and Avg DEC_E_ presenting moderate levels of agreement. However, some caution may be advised with regards to TTS and DEC Dist as in other studies, these metrics have been found to produce poor to moderate levels of inter-day reliability (8) and elevated within-session variability scores (12, 21). Additionally, only showing moderate levels of agreement, Avg DEC_E_ presented with the greatest inter-day CV%, suggesting that between raters up to 7.8% of variability may be seen due to differences in the manual data processing procedures. Avg DEC_E_ being the average change in velocity between Max Velo and 50% of Max Velo, it is possible that the lack of clear guidelines or thresholds for removal of outliers around the top of the velocity-time curve during manual data processing may be responsible for the reported variability. It seems that this location on the velocity-time curve represents a window in which individual interpretations may negatively influence the consistency between raters. Readers may refer to Figure 1 which presents a visualization of an unprocessed and processed velocity-time curve in which the removal of a single outlier affects the point of Max Velo used to determine Avg DEC_E_. More specifically, this figure was modified to only show the acceleration and the deceleration phase, and not the phase of the backpedal. With this in mind, defining the start of the deceleration phase as the first instantaneous velocity point following the athletes’ maximal approach velocity may fail to take into account the athletes' “coasting” or “transitional” phase prior to increasing their rate of deceleration. Future research may explore the utility of other start of deceleration phase detection thresholds. While speculative, jerk being the derivative of acceleration/deceleration with respect to time may offer additional insights into the identification of different kinematic events and phases, similar to research looking at the yank-time signal in vertical jumps (31).

Furthermore, assessing the intra-rater (i.e., within-session, test-retest) reliability and variability analyses, across all four raters, Avg DEC_L_ showed poor levels of consistency between trials. This suggests the later parts of the deceleration phase may be prone to biological and performance related variability, while the early deceleration phase may be more impacted by rater-related differences in the manual processing procedures. TTS and DEC Dist also presented with ICCs below 0.50, suggesting poor consistency between the two ADA test trials. Given that they reflect the time and distance when decelerating to a stop, the previous two metrics may carry ecological validity regarding the communication with coaches and non-sport science practitioners. However, their questionable intra-rater reliability and variability shown across different studies raises concerns with regards to their utility with athlete populations. While speculative, the low degrees of reliability with regards to DEC Dist may be influenced by the absence of a predetermined stopping point for the deceleration phase. In the ADA, athletes are instructed to rapidly decelerate at a specific location, however the end of the deceleration phase is not provided. Furthermore, while instructed to initiate the deceleration phase as close to the 9.14-m marker as possible, athletes may initiate the deceleration phase prior to or after the 9.14-m marker. To avoid such pacing strategies, previous research has implemented possible solutions, such as comparing athlete's approach sprint times during the ADA to their times during a maximal linear sprint test over the same distance and eliminating trials if the difference between the two is above or below a selected threshold (8). However, in applied settings with large groups of athletes being tested at the same time, these solutions may be challenging to adopt. When looking at the sprint acceleration phase of the ADA (i.e., Avg ACC, Max ACC, Max Velo), Avg ACC presented with excellent levels of consistency across all four raters, while Max Velo showed moderate levels of consistency, and Max ACC showed poor to moderate levels of consistency. In both the inter-rater and intra-rater analyses, Avg ACC, Avg DEC showed greater ICCs compared to Max ACC, Max DEC. This has been shown in previous research suggesting better reliability and sensitivity with taking the absolute value of all raw acceleration and deceleration values and averaging them over the duration of a selected time period (32, 33). Limitations with calculating maximal vs. average metrics should be taken into consideration when interpreting these data.

**Figure 3:**
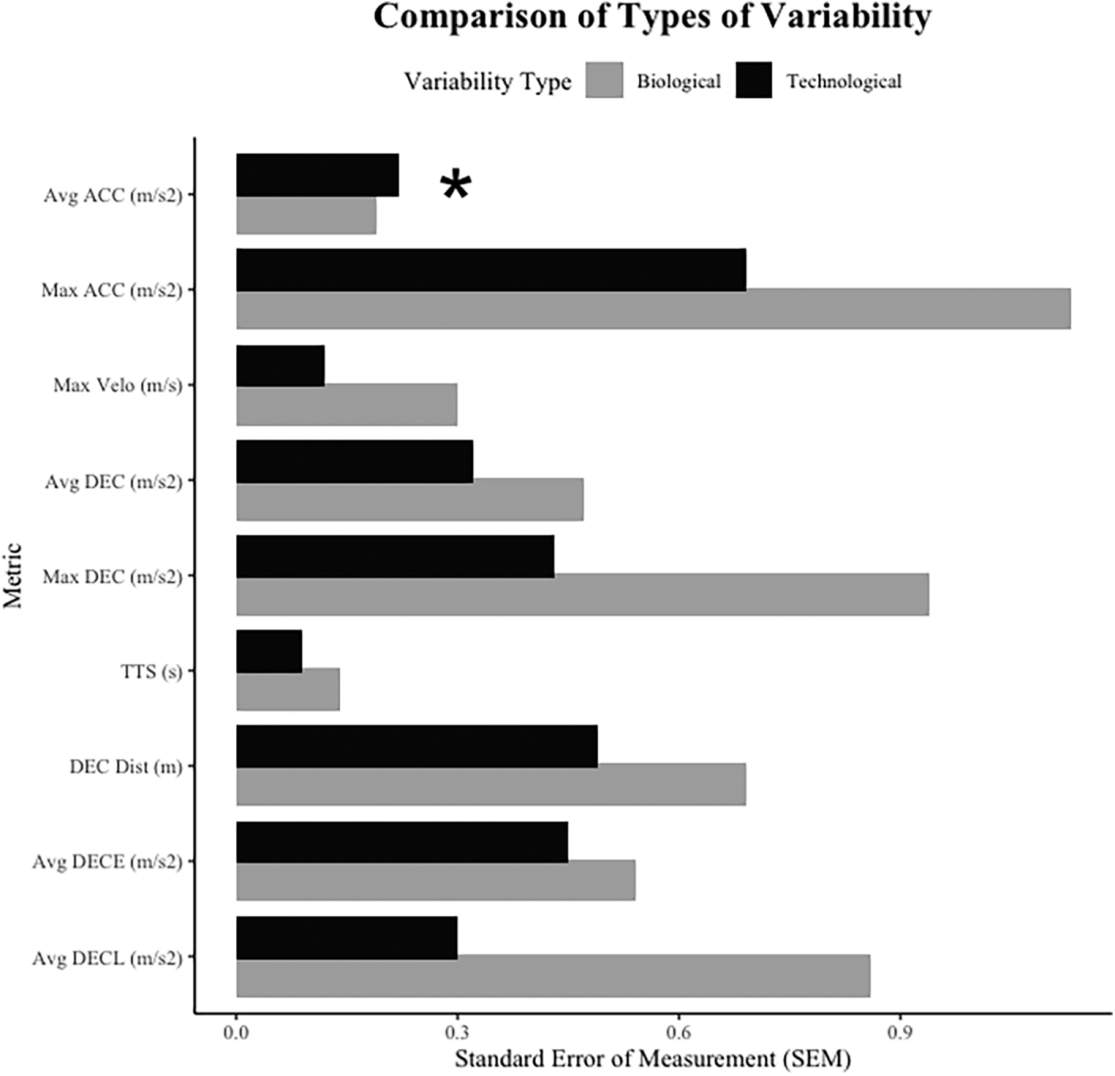
Comparison between technological variability and biological variability reported in this study's results. Inter-rater SEM values reflect technological variability, while intra-rater (average across four raters) SEM values reflect biological variability. *“*” suggests that technological variability was found to be greater than biological variability.

Additionally, readers should appreciate the differences in the types of variability presented in in this study. Intra-rater variability may be thought of as biological variability influenced by intra-athlete differences in performance between trials, while inter-rater variability may be thought of as technological variability, influenced by the between-rater differences related to the manual and somewhat arbitrary nature of the data processing procedures. While some biological variance is to be expected, technological variability should not exceed the variability induced by biological factors. Figure 3 presents a comparison for metric-specific SEM values, comparing biological and technological variability in our data. Figure 3 shows that biological variability exceeds technological variability for all metrics except for Avg ACC, which while marginally different, showed greater technological variability. Similar to earlier discussions about different locations along the velocity-time curve, the start of each ADA trial may also present a window for individual interpretation of about outlier removal and initiation of the start of the trial. While speculative, our data suggests that manual processing procedures at both the start of each trial, as well as around the top of the velocity-time curve (i.e., start of deceleration) show the largest windows for individual rater interpretation, which could induce unnecessary variability into ADA-derived acceleration and deceleration metrics. Ultimately, this process should become fully automated, based on selected filters or algorithms, rather than the subjectivity of the rater.

To the authors' best knowledge, this is the first study attempting to assess the between-rater agreement for processing and assessing maximal horizontal deceleration performance using radar technology in athletes. Furthermore, authors believe to date, this is the largest sample of NCAA division 1 athletes performing the maximal horizontal deceleration assessment used in this study. However, limitations with the ADA and future avenues of research should still be acknowledged. In our study descriptive statistics for both American football, and Lacrosse athletes suggested that most athletes required between 4.5 and 5 m to maximally decelerate. This is in line with findings by Graham et al. who investigated athletes' deceleration ability in relation to their self-determined limit to accelerate over different prescribed distances (9). In this study, it took athletes 4.94 ± 0.39 m to decelerate to a stop, following a 10-m acceleration during which they achieved 72.2 ± 3.2% of their maximal speed (30-m maximal sprint). It could be worthwhile for sport-science practitioners to further look into deceleration qualities during tasks in which a distinct stopping or turning point is identified. Further, athletes requiring between 4.5 and 5 m to maximally decelerate following a 10-m sprint seems to match up well with the dimensions of the 5-0-5 change of direction deficit test, which could give a more holistic insight into not only horizontal deceleration ability, but also the ability to efficiently turn and re-accelerate. Using radar-, or laser-technology to quantify deceleration and re-acceleration qualities in a task such as the 5-0-5, similar to more recent research (12) may productively add to the existing body of evidence with regards to adding context to deceleration demands and braking strategies during change of direction maneuvers.

Likely the overarching strength of this study is the size and trained nature of the population investigated, as well as the fact that both males and females were studied. The breadth and depth of descriptive information provided in this study may also allow sport science practitioners to use results in comparing or benchmarking athletes. Authors believe this to be the largest sample of trained athletes, both male and female performing the novel, maximal horizontal deceleration task used in this study. Otherwise, limitations pertaining to this study may be identified in the applied nature of the investigation. When working with high-level collegiate athletes, researchers often struggle to control for outside variables such as sleep, nutrition, as well as hydration, amongst others. Future studies may aim to replicate methodologies implementing more rigor with regards to control for outside factors potentially affecting athlete performance. Additionally, future research investigating the intra-rater reliability of deceleration measures may decide to include more than two test trials for each athlete, and further investigate the variation in selected metrics between test-days. Furthermore, future studies may replicate procedures across additional populations, and deceleration assessments, allowing for greater generalization of our findings. Regardless, given the substantial sample size of high-level male and female athletes in studying the reliability of an emerging physical construct of horizontal deceleration performance, the authors believe that this study effectively contributes to a growing body of literature.

This study documented moderate to excellent levels of agreement between four individual raters in quantifying maximal horizontal deceleration performance within the ADA test. Based on our data, it may be speculated that if a foundational understanding and agreement of manual data processing procedures for radar-derived data is given between raters, metrics may be interpreted with moderate to excellent levels of confidence. However, when possible, and when using the Stalker ATS radar technology, authors advise practitioners to use one trained individual to manually process raw data. Ideally, this process should become fully automated, based on selected filters or algorithms, rather than the subjectivity of the rater. Particular caution in the manual data cleaning process may be used for Avg ACC and Avg DEC_E_, as based on our data, these metrics present with more variability between raters. This is likely influenced by the processing procedures around the start of the trial and the top of the velocity-time curve (i.e., start of deceleration), which seem to leave a greater window for individual interpretation. The intra-rater analyses revealed poor to excellent consistency between ADA trials, with CV%'s ranging from 3.1% to 13.2%, depending on the respective metric and rater. Caution may be advised with regards to the intra-rater reliability of TTS and DEC Dist across different ADA trials. Findings presented in this study may be of acute interest to sport science practitioners working with multi-directional sport athletes and interested in reliably quantifying maximal horizontal deceleration performance.

## Data Availability

The raw data supporting the conclusions of this article will be made available by the authors, without undue reservation.
